# Effect of Essential Oils on Postharvest Management of Anthracnose Associated with *Colletotrichum gloeosporioides* (Penz.) Penz & Sacc., in Mango

**DOI:** 10.3390/plants14213249

**Published:** 2025-10-23

**Authors:** Petra Andrade-Hoyos, Marianguadalupe Hernández-Arenas, Aarón Mendieta-Moctezuma, Edwin Javier Barrios-Gómez, Omar Romero-Arenas, Selma Ríos-Meléndez, Conrado Parraguirre-Lezama, Patricia Ibarra-Torres

**Affiliations:** 1 Instituto Nacional de Investigaciones Forestales Agrícolas y Pecuarias (INIFAP), Campo Experimental Zacatepec, Carretera Zacatepec-Galeana s/n, Km 0.5, Colonia IMMS, Zacatepec 62780, Morelos, Mexico; petra.andrade.hoyos@gmail.com (P.A.-H.); hernandez.marian@inifap.gob.mx (M.H.-A.); barrios.edwin@inifap.gob.mx (E.J.B.-G.); 2 Centro de Investigación en Biotecnología Aplicada, Instituto Politécnico Nacional, Carretera Estatal Santa Inés Tecuexcomac-Tepetitla, Km 1.5, Tepetitla de Lardizábal 90700, Tlaxcala, Mexico; sriosm@ipn.mx; 3 Centro de Agroecología, Instituto de Ciencias, Benemérita Universidad Autónoma de Puebla, Edificio VAL 1, Km 1.7 Carretera a San Baltazar Tetela, San Pedro Zacachimalpa 72960, Puebla, Mexico; conrado.parraguirre@correo.buap.mx; 4 Ingeniería en Biotecnología, Universidad Politécnica de Guanajuato, Avenida Universidad Sur 1001, Localidad Juan Alonso, Cortazar 38496, Guanajuato, Mexico; pibarra@upgto.edu.mx

**Keywords:** control, anthracnosis, *Colletotrichum gloeosporioides*, post-harvest, mango

## Abstract

This study evaluated the efficacy of essential oils (EOs) for the postharvest management of anthracnose caused by *Colletotrichum gloeosporioides* in mango. EOs from Cinnamon (*Cinnamomum zeylanicum*), Oregano (*Origanum vulgare*), Rosemary (*Salvia rosmarinus*), and Black pepper (*Piper nigrum*) were chemically characterized using gas chromatography–mass spectrometry (GC–MS). The main compounds identified included eugenol, methyl eugenol, carvacrol, and eucalyptol, all recognized for their antifungal and antioxidant properties. In vitro assays showed that cinnamon and black pepper EOs significantly inhibited mycelial growth of *C. gloeosporioides* at all tested concentrations, whereas rosemary EO exhibited lower efficacy. In vivo experiments confirmed that all tested EOs significantly reduced disease severity in mango fruits compared to the control. Overall, the antifungal activity of EOs was dose-dependent and strongly influenced by their chemical complexity and synergistic interactions among major and minor components. These findings highlight the relevance of oxygenated monoterpenes, hydrocarbon monoterpenes, and sesquiterpenes as candidate groups for developing sustainable alternatives for the control of *C. gloeosporioides* in mango production.

## 1. Introduction

Mexico ranks fifth globally in mango (*Mangifera indica* L.) production volume, following India, Indonesia, China, and Pakistan. In the 2023 production year, Mexican mango exports reached approx. 450,000 tons, generating around USD 575 million, with over 90% of this value attributed to the U.S. market [1]. The country also achieved a cultivated area of almost 219,500 hectares, with an average yield of 10.85 tons per hectare, reflecting a trend of sustained growth in recent years, recognizing that mango production plays a key role in the generation of employment and currencies. This productive boom is concentrated in five states—Sinaloa, Guerrero, Nayarit, Chiapas, and Oaxaca—which collectively contribute over 76% of the national output [2]. However, mango cultivation continues to face significant challenges that affect fruit quality and yield.

One of the major issues in mango production is the incidence of diseases caused by phytopathogens throughout the crop cycle. These diseases can affect various plant organs, including stems, roots, branches, leaves, flowers, and fruits. The postharvest stage is particularly critical, as it results in significant commercial and economic losses due to fruit rot and lesions [3]. These issues are exacerbated by the prevailing climatic conditions in production regions, which favor pathogen proliferation [4].

Once harvested, mango fruits become especially vulnerable to infections due to a decline in natural resistance after detachment from the mother plant [5]. Furthermore, their high moisture and sugar content create a favorable environment for the activation of pathogens that remained latent during development [6]. This situation poses a major challenge for product preservation and marketing, particularly when meeting international quality standards for export.

In Mexico, the most significant mango disease is anthracnose, caused by *Colletotrichum* spp., which leads to estimated yield losses of 5–15%—equivalent to 4.5 to 13.5 tons per hectare [7]. This disease not only reduces marketable yield but also affects fruit quality, decreasing its commercial value and international market acceptance [8]. Thus, anthracnose is a major limiting factor for the global competitiveness of Mexican mango. Anthracnose can occur at various crop stages. When inflorescences are infected, yields are drastically reduced, particularly in poorly managed orchards. In immature fruits, the infection remains quiescent due to the presence of antifungal compounds known as dienes, which are localized in the fruit’s exocarp [9]. These compounds inhibit fungal colonization during early fruit development [10]. However, as the fruit ripens, diene concentrations decline, enabling fungal hyphae to penetrate and colonize the cell wall, resulting in disease development.

The reproduction and severity of the pathogen are strongly influenced by environmental factors such as temperature and humidity, which vary by production region. As a climacteric fruit, mango is highly susceptible to *Colletotrichum* infection, making it a persistent concern for growers and exporters [10]. During postharvest, anthracnose is mainly expressed as black or dark spots on the surface, negatively impacting its appearance and commercial value. To reduce disease risk, chemical fungicides are commonly applied. These are regulated by importing countries to ensure food safety and product acceptability [11]. However, excessive or improper use of these chemicals may result in maximum residue level (MRL) violations, leading to trade sanctions and economic losses [12]. Moreover, *C. gloeosporioides* has increasingly developed resistance to several fungicides due to its high adaptability and genetic variability, complicating control through conventional methods [13,14].

Given these limitations, scientific research has focused on more sustainable and environmentally friendly alternatives for postharvest disease management. Among these, plant extracts and EOs have gained increasing attention due to their antifungal, antibacterial, and antioxidant properties, which are largely attributed to aromatic compounds and phenolic groups. These compounds have demonstrated efficacy in inhibiting pathogenic fungal growth in both in vitro studies and practical applications [15]. Additionally, EOs are biodegradable and exhibit low or no toxicity, making them an eco-friendly option for disease control in fruits and vegetables [16].

Recent studies have demonstrated the effectiveness of EOs such as cinnamon (*C. zeylanicum*), oregano (*O. vulgare*), rosemary (*S. rosmarinus*), and black pepper (*P. nigrum*) in controlling postharvest pathogens. For instance, Cinnamon EO—rich in eugenol and cinnamaldehyde—has shown significant inhibition of *C. gloeosporioides* in stored mangoes, reducing anthracnose incidence by up to 80% [17]. Similarly, oregano EO, which contains high levels of carvacrol and thymol, has displayed strong antifungal activity against *Botrytis cinerea* in table grapes, extending shelf life without synthetic residues [18]. Rosemary EO, known for its terpene content, has proven effective against *Penicillium expansum* in apples by reducing blue and green mold occurrence during cold storage [19]. Black pepper EO has also shown notable fungal load reduction in papayas and bananas infected with *Aspergillus niger*, without altering the fruits’ organoleptic properties [20]. The application of EOs offers the potential to extend shelf life, maintain fruit quality, and reduce dependency on synthetic fungicides—important steps toward more sustainable and safer postharvest practices in horticultural production [4,21]. The present work aims to evaluate and characterize the effectiveness of four EOs in the inhibition of the growth of *C. gloeosporioides* through in vitro tests, as well as to determine its effect on the reduction in the severity of anthracnosis in mango fruits treated in post-harvest conditions, in order to identify viable natural alternatives for the management of this disease.

## 2. Results

### 2.1. EOs Analysis

Gas chromatography-mass spectrometry (GC-MS) analyses of EOs extracted by steam distillation from *C. zeylanicum* Blume., (Cinnamon), *S. rosmarinus* Spenn., (Rosemary), *O. vulgare* L., (Oregano) and *P*. *nigrum* L., (Black pepper) showed varied and complex chemical profiles (Figure S1 and Table 1), with yields between 0.36% (Cinnamon), 0.8% (Oregano), 0.55% (Black pepper) and 2.0% (Rosemary). In cinnamon EO, the main compounds were eugenol (61.88%), benzyl alcohol (14.44%), linalool (3.49%) and cinnamaldehyde (2.97%), highlighting eugenol as a key bioactive metabolite. Rosemary EO presented a profile dominated by eucalyptol (32.42%) and α-pinene (30.78%), along with verbenone (7.04%), myrcene (4.58%), and borneol (3.99%), reflecting its characteristic complexity. In oregano, hydrated sabinene (21.68%), carvacrol (6.75%), and thymol methyl ether (4.62%) were identified, with a total of 85.03% volatile compounds. Finally, the black pepper showed methyl eugenol (46.28%), eugenol (32.93%), β-myrcene (9.54%), and eucalyptol (4.33%) with 98.69% of identified compounds. These results confirm chemical variability according to species and regions and highlight the importance of phenols and terpenoids in the biological properties of EOs [22,23].

The differential chemical wealth observed in EOs supports its potential for biological applications, especially in the control of post-harvest pathogens, thanks to the presence of secondary metabolites with antifungal, antibacterial and antioxidant activity. For example, the high concentration of eugenol and methyl eugenol in *P. nigrum* and *C. zeylanicum* (Figure S2), coincides with reports that attribute to these compounds’ powerful antimicrobial effects [24]. Likewise, the abundance of eucalyptol and α-pinene in *S. rosmarinus* is related to recognized antioxidant and antimicrobial properties [25]. The complex composition of Oregano EO, with carvacrol and sabinene hydrated as main components, reinforces its traditional and scientific use as a natural antifungal agent. Comparative analysis of the volatile composition of EOs reveals a marked chemical diversity among the studied species. In the case of *S. rosmarinus*, a high concentration of bicyclic monoterpenes was identified, particularly α-pinene (30.78%) and 1,8-cineole or eucalyptol (32.42%), respectively (Figure S3).

These compounds are widely recognized for their antioxidant and antimicrobial effects, as indicated by Leesutthiphonchai et al. [24]. This chemical composition suggests a relevant preservative and therapeutic functional profile, according to what is reported by Sun et al. [26], who highlights the close relationship between the cyclic ether and biological activity in species of the *Lamiaceae* family.

*O. vulgare* showed a remarkable chemical complexity, highlighting a high proportion of hydrated sabinene (21.68%) and the significant presence of phenols such as carvacrol (6.75%), as well as other monocyclic monoterpenes such as γ-terpinene and α-terpinolene. This composition confers carvacrol a high antifungal and antioxidant potential, according to Danh [23], altering the permeability of the cell membrane in microorganisms, while other monoterpenes exert synergistic effects that expand their antimicrobial spectrum. According to Vilela [27], EO rich in alcohols and phenols, such as those present in *O. vulgare*, not only inhibit pathogens, but also protect plant tissues against oxidative stress, being relevant for post-harvest applications. Together, the high proportion of oxygenated compounds in *O. vulgare* supports its effectiveness as a biocontrol agent, with potential application in food conservation through sustainable and natural strategies.

**Figure 1:**
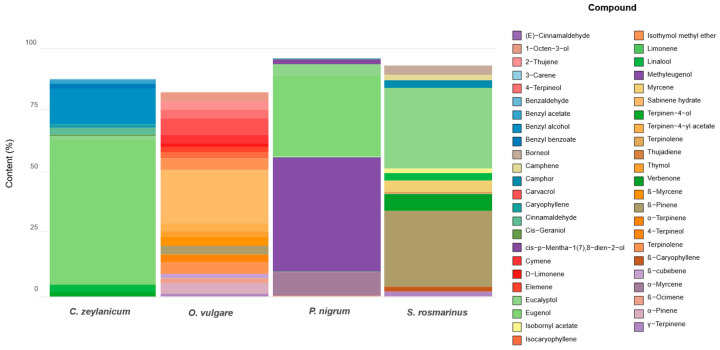
Global chemical profile of EOs: distribution of compounds in the species analyzed.

As for *P. nigrum*, this EO was characterized by a marked predominance of 79.21% of aromatic terpenes (methyl eugenol (46.28%) and eugenol (32.93%)) as major compounds (Figure 1). Although monoterpenes and sesquiterpenes were also detected as β-myrcene (9.54%), limonene (0.57%), β-ocimene (0.75%), and β-caryophyllene (0.82%), these are found in lesser proportion. β-Caryophyllene, in particular, is a sesquiterpene with widely described anti-inflammatory effects [28]. This composition gives *P. nigrum* EO a bioactive profile with an emphasis on antimicrobial properties, although less diverse in terms of functional groups compared to *O. vulgare* EO.

For its part, the EO of the leaf of *C. zeylanicum* presented a profile dominated by the phenylpropanoid eugenol (61.88%) as the majority compound, followed by benzyl alcohol (14.44%) and cinnamaldehyde. This richness in phenolic compounds (Figure 1) coincides with that reported by Leesutthiphonchai et al. [24], who describes eugenol as a potent antimicrobial and antioxidant agent. The presence of aromatic aldehydes, such as cinnamaldehyde, reinforces its therapeutic potential, providing this EO with a highly bioactive functional profile.

The functional distribution of EOs, analyzed from the perspective of organic chemistry, shows that biological properties are strongly determined by the predominant functional groups. Thus, aromatic monoterpenoids are associated with high antimicrobial and antioxidant capacity, while oxygenated monoterpenes and sesquiterpenes are related to anti-inflammatory and preservative effects [26,27,28]. This analysis confirms that the variability in the chemical composition of EOs is determined by genetic, environmental, and methodological factors, which influence both the identity of volatile compounds and their biological functionality. Intra- and inter-population genetic diversity modulates the biosynthetic capacity of plants to produce secondary metabolites, such as monoterpenes, sesquiterpenes, and phenols, giving rise to different chemotypes even within the same species. In the case of *O. vulgare*, studies have shown that genetic variability is associated with differences in the proportion of key compounds, such as carvacrol and thymol, which directly impacts the quality and biological efficacy of the EOs [29].

**Figure 2:**
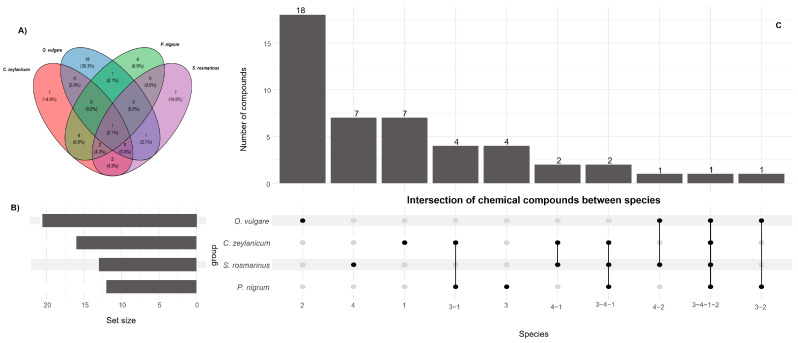
Distribution of metabolites in the plant species studied. (**A**) Terpenes shared between the four EOs evaluated; (**B**) Intrinsic diversity of each essential oil; and (**C**) Diversity of chemical classes of EOs analyzed.

The intersection analysis of volatile compounds (Figure 2A) revealed the presence of shared metabolites among the four evaluated species. Notably, α-pinene was ubiquitous, detected in all species with particularly high abundance in *S. rosmarinus* (30.78%). Likewise, eucalyptol was found in three species (*P. nigrum*, 4.33%; *S. rosmarinus*, 32.42%; *C. zeylanicum*, 1.39%), along with linalool (*P. nigrum*, 0.51%; *S. rosmarinus*, 2.91%; *C*. *zeylanicum*, 3.49%) and limonene (*P. nigrum*, 0.57%; *C. zeylanicum*, 0.72%; *O. vulgare*, 1.24%). These overlaps reflect a shared pattern of oxygenated and hydrocarbon monoterpenes (Table 1), functionally associated with antioxidant and antimicrobial properties [30].

Specific intersections between pairs of species were also observed, including verbenone (7.04% in *S. rosmarinus* and 1.55% in *C. zeylanicum*), camphor (3.07% and 0.45%, respectively), and γ-terpinene (1.93% in *S. rosmarinus* and 4.61% in *O. vulgare*). In contrast, β-caryophyllene, a common sesquiterpene in essential oils, was found in three species (*P*. *nigrum*, 0.82%; *S. rosmarinus*, 2.05%; *C. zeylanicum*, 0.68%), indicating a relevant contribution of bicyclic sesquiterpenes to their overall composition.

Metabolic divergence was particularly evident in *C. zeylanicum* and *P. nigrum*, where oxygenated aromatic monoterpenes reached 84.15% and 79.55%, respectively. The predominance of eugenol (61.88% in *C. zeylanicum*, 32.93% in *P. nigrum*) and methyl eugenol (46.28% in *P. nigrum*) defines a distinctive phenolic profile consistently associated with broad-spectrum antimicrobial mechanisms [31,32].

From a functional perspective, *S. rosmarinus* exhibited a predominance of oxygenated bicyclic monoterpenes (82.36%), mainly eucalyptol and α-pinene, compounds linked to its well-known antioxidant and preservative properties [33,34]. In *O. vulgare*, the dominant fraction corresponded to bicyclic (34.85%) and monocyclic monoterpenes (17.2%), with major compounds such as carvacrol (6.75%), γ-terpinene (4.61%), and sabinene hydrate (21.68%), suggesting a mixed profile of hydrocarbon and oxygenated alcohol monoterpenes of biological relevance [30].

The intrinsic diversity of each essential oil (Figure 2B, Table 1) revealed significant differences in chemical composition. *O. vulgare* exhibited the highest number of exclusive compounds (18), organized within a profile dominated by bicyclic and monocyclic monoterpenes. In contrast, *C. zeylanicum* and *S. rosmarinus* each contained seven unique compounds, while *P. nigrum* had the fewest (four). This distribution reflects the metabolic specialization of each species: while *S. rosmarinus* and *O. vulgare* concentrate oxygenated monoterpenes, *P. nigrum* and *C. zeylanicum* stand out for their high content of aromatic phenols, resulting in distinct bioactive and therapeutic profiles (Figure 2B).

The analysis of chemical classes (Figure 2C, Table 1) revealed a structural and functional complementarity among the evaluated chemical systems. A fundamental dichotomy was identified between oils dominated by non-aromatic oxygenated monoterpenes (*S. rosmarinus*, *O. vulgare*) and those specialized in aromatic phenols (*C. zeylanicum*, *P. nigrum*). The presence of hydrocarbon sesquiterpenes (β-caryophyllene, β-cubebene) in proportions ≤4% across all species, together with specific intersections such as verbenone and γ-terpinene, reinforces the existence of divergent biosynthetic strategies with distinct functional implications.

These findings support the potential for the design of synergistic mixtures that exploit the functional specialization of each chemical profile, particularly for postharvest pathogen control, where the complementarity of action mechanisms could overcome the limitations of individual compounds. Overall, the chemical diversity observed among the evaluated species highlights their potential as biocontrol agents, due to the presence of metabolites with antifungal, antibacterial, and antioxidant activities. In particular, the high concentrations of eugenol and methyl eugenol in *P. nigrum* and *C. zeylanicum*, as well as eucalyptol and α-pinene in *S. rosmarinus*, explain their proven biological efficacy, while the chemical complexity of *O. vulgare*, dominated by carvacrol and sabinene hydrate, reinforces its value as a natural antifungal agent.

### 2.2. Antifungal Tests

**Figure 3:**
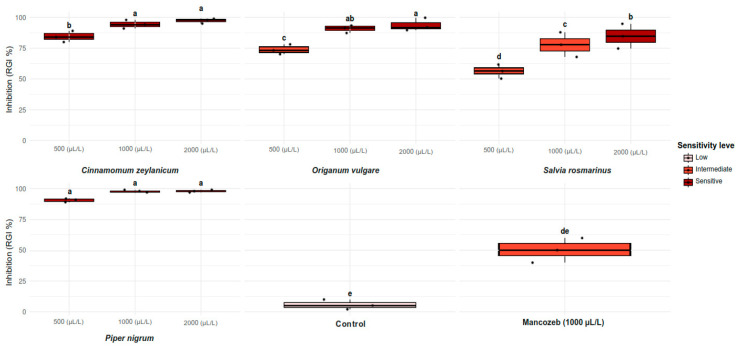
Percentage of mycelial growth inhibition of *C. gloeosporioides* in different concentrations of EOs of four plant species. Equal letters indicate that there is no significant statistical difference between treatment (*p* = 0.05). RGI = percentage of mycelial growth inhibition. Mancozeb = chemical witness, control = distilled water.

The inhibition of the growth of the MA-L4 strain of *C. gloeosporioides* by the EOs evaluated showed variability depending on the type of EO and the applied concentration, observing a directly proportional relationship between concentration and inhibition. *P. nigrum* EO (Black pepper) achieved a complete inhibition of the pathogen in all proven concentrations (Figure 3), the same results were achieved with the two highest concentrations in cinnamon EO. In contrast, *S. rosmarinus* EO (Rosemary) presented the lowest efficacy, with 56.7% of inhibition to the lowest concentration, like the chemical witness, without significant statistical differences between both treatments.

These results are consistent with the wide range of antimicrobial activities reported for the genus *Piper*, which include medical and agricultural applications for the control of pests and diseases, attributed to its chemical diversity [35,36,37]. Previous studies have demonstrated the efficacy of *P. macedoi* EO in the inhibition of the development of *Colletotrichum musee* up to 90% to specific concentrations [38]. Additionally, other EO such as *Conyza bonaerensis* have shown inhibitory effects on mycelial growth and damage to the cell membrane in insulation of *Colletotrichum* spp. [39].

**Figure 4:**
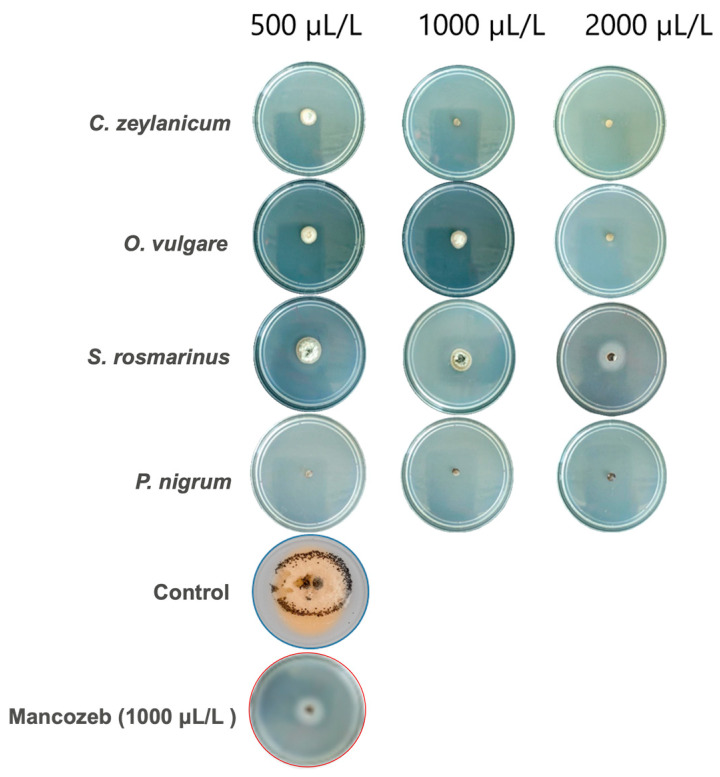
Inhibition of *C. gloeosporioides* in the middle of culture Potato Dextrose Agar (PDA) with four plant species to three different concentrations of EOs. Mancozeb = positive control, Distilled water = negative control.

Although *S. rosmarinus* showed less activity in this study, previous investigations report total control of the mycelial growth of *Colletotrichum lindemuthianum* with EO of this species, which suggests that the sensitivity of the pathogen can vary according to the specific species involved [40]. Finally, the sensitivity of the pathogen to the different concentrations of EOs correlated with the inhibition levels observed (Figure 4), confirming the dose–response dependence on the antifungal activity.

The sensitivity of the pathogen is directly related to the type of EOs that interacts with the pathogen, since they present various control mechanisms due to the compounds that make it up, mainly the integrity of the cell wall is compromised by interference in the synthesis of lipids in the cell wall, there is lysis and consequently the death of the cell [41].

The intersection analysis of volatile compounds provides valuable insights into these differences. The ubiquity of α-pinene across all EOs, with particularly high abundance in *S. rosmarinus* (30.78%), did not translate into superior antifungal activity in our assays, suggesting that inhibition is not exclusively determined by the presence of single compounds but rather by their concentration thresholds, synergistic effects, and interactions with other metabolites. Similarly, eucalyptol, linalool, and limonene— oxygenated and hydrocarbon monoterpenes—were shared across multiple EOs, evidencing a common chemical pattern among these species. The distribution of sesquiterpenes such as caryophyllene, present in *P. nigrum*, *S. rosmarinus*, and *C*. *zeylanicum*, is particularly relevant, given that these compounds have been associated with membrane disruption and antifungal activity in several phytopathogenic fungi.

### 2.3. Fresh Fruit Severity Tests

**Figure 5:**
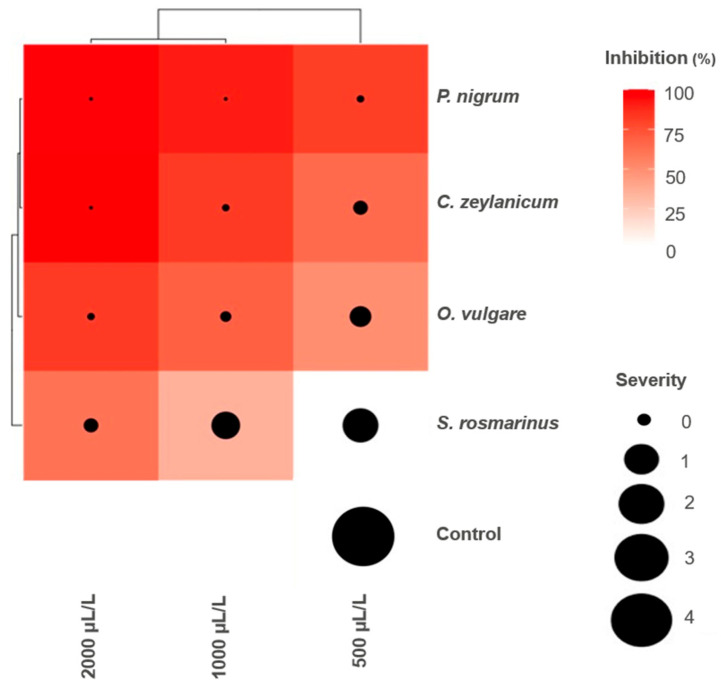
Inhibition of the severity of *C. gloeosporioides* in mango fruits treated with variable doses of EOs of four plant species. Distilled water = negative control.

**Figure 6:**
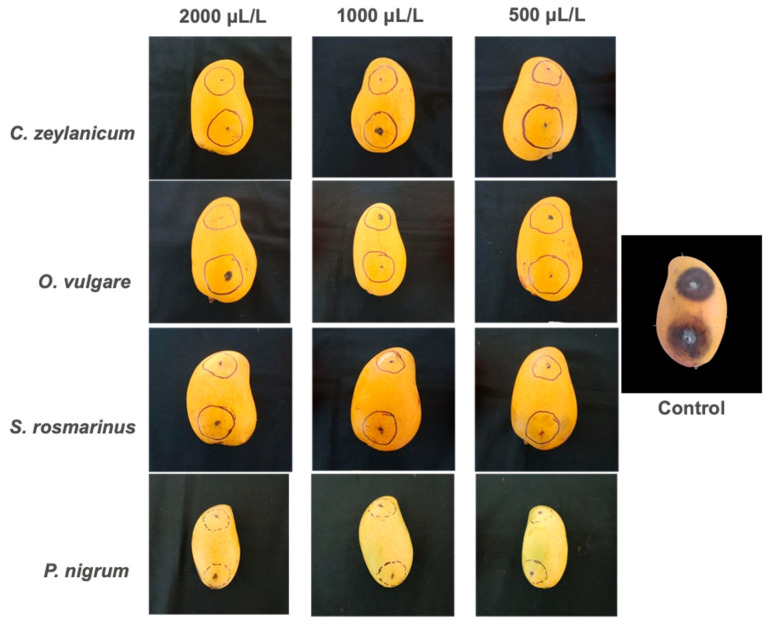
Mango fruits showing the damage and severity caused by *C. gloeosporiodes* under treatments based on EOs. Negative control = Distilled water.

In the present study, EOs showed superior performance in the bioassay in vivo compared to the in vitro test, as evidenced in Figure 5 and Figure 6. Significant statistical differences were observed only between treatments with EOs and the absolute witness, which confirms the antifungal effect of all the EOs evaluated against *C. gloeosporioides*. As for the severity of the disease, all treatments maintained healthy fruits, except treatment with *S. rosmarinus* in its lowest concentration and the absolute witness (Figure 6). The highest severity values were recorded in *C. zeylanicum* in the three concentrations evaluated, while in the in vitro test, *P. nigrum* showed the greatest inhibition. It should be noted that treatments with *S. rosmarinus* presented a better control in vivo conditions than in vitro, with a difference in inhibition of up to 27.24% in their lowest concentration (Figure 5).

These findings contrast with those reported by Maqbool et al. [42], who indicated that the EOs of lemon and cinnamon were more effective in vitro than in applications on papaya and bananas fruits. In this work, the minimum damage observed in the fruits treated, even with the lowest concentration of *S. rosmarinus*, was classified as “traces” of disease (Figure 6), which supports the effectiveness of EOs in post-harvest control. Previous studies have demonstrated the potential of EOs to control various post-harvest diseases, including those caused by *Colletotrichum* spp. [27,43,44,45]. For example, [46] reported that *C. zeylanicum* EO reduces severity in mango fruits, compared to fruits without treatment.

In addition, *P. nigrum* has been identified as a promising EO for the control of pathogens of the genus *Colletotrichum*, which shows its powerful antifungal activity [47]. For example, a study published in Food Science and Technology showed that the nanoemulsion of black pepper EO significantly inhibits the growth of *C*. *gloeosporioides* by negatively affecting the respiratory metabolism of the fungus, reducing by more than 60% its respiratory rate by suppression of key enzymes in the cycle of tricarboxylic acid and the pathway EMBDEN-Meyerhof-Parnas [48]. This biochemical mechanism highlights the potential of EOs to mitigate the proliferation and reproduction of the pathogen.

The strong inhibitory effect of *P. nigrum* observed in this study may be attributed not only to the abundance of certain volatiles but also to the synergistic contribution of its diverse chemical profile, which has been previously reported for its broad antimicrobial properties in both agricultural and medical contexts [35,36,37]. Conversely, although *S. rosmarinus* has been shown in previous studies to fully inhibit *C. lindemuthianum* [40], its lower activity against *C. gloeosporioides* MA-L4 suggests that antifungal sensitivity is strongly species-dependent, reaffirming the importance of pathogen-specific evaluations.

Likewise, research reported in Journal of Science and Agricultural Technology have confirmed the effectiveness of raw extracts of various species of the *Piper* genus, including *P. nigrum*, to inhibit the mycelial growth of *Colletotrichum capsici*, causal agent of anthracnosis in Chile. The phytochemical compounds present, such as flavonoids, phenols, alkaloids and terpenoids, contribute to this antifungal activity, showing an inhibition superior to conventional positive controls [47]. Reported that *Pimenta dioica* achieved total control (100%) of severity in pepper fruits [49]. Together, these results suggest that EOs constitute an effective alternative for the management of post-harvest diseases associated with the genus *Colletotrichum*, reinforcing its relevance in biocontrol strategies.

## 3. Materials and Methods

### 3.1. Biological Material

This study worked with the *C. gloeosporioides* (Penz.) Penz & Sacc., strain MA-L4, previously molecularly characterized by Sanger sequencing of the internal transcribed spacer (ITS) region (GenBank accession number: OR466677.1), using the ITS1 and ITS4 primer pair. The strain MA-L4 is protected at the Centro de Agroecología-ICUAP de la Benemérita Universidad Autónoma de Puebla, Mexico. The isolation comes from the variety of “lemon” that infects Persian files in Mexico, which guarantees the identity and specificity of the pathogen used for experimental tests.

### 3.2. Obtaining EOs

Fresh *C. zeylanicum* Blume., and dry fruit of *P. nigrum* L., were collected in the local market of Cuetzalan, Puebla-Mexico, the fresh plants of *O. vulgare* L., and *S. rosmarinus* Spenn., belong to the municipality Ixtacuixtla, Tlaxcala-Mexico. The plant material was dehydrated at room temperature in the shade for two weeks before processing [50]. Its taxonomy was also corroborated at the TLMX Herbarium (voucher numbers 9472–9475), located in Tlaxcala City, Mexico.

EOs was obtained from dehydrated leaves of C. zeylanicum, O. vulgare and S. rosmarinus as well as from the dried fruit of P. nigrum using twenty kilograms of plant material of each species by steam distillation using a stainless-steel distillation apparatus (essential distiller, Inoximexico, Guadalajara, Mexico) for 3 h. EOs samples were dehydrated with anhydrous sodium sulfate and stored in amber glass bottles at 4 °C [51]. The chemical characterization of the metabolites present in the EOs was carried out by Gas Chromatography (Clarus 580, PerkinElmer, Inc., Waltham, MA, USA) coupled to Mass Spectrometer (Clarus SQ8S, PerkinElmer, Inc., Waltham, MA, USA) (GC-MS) equipped with an Elite-5MS column (PerkinElmer, Inc., Waltham, MA, USA) (30 m × 0.32 mm, film thickness 0.25 µm) and interfaced with the NIST Mass Spectral Search Program (software version 2.0). Kovats retention index were determined relative to the retention time of a homologous series of n-alkanes (C8-C20) (Sigma-Aldrich, St. Louis, MO, USA) analyzed under the same conditions [52].

### 3.3. In Vitro Evaluation of Antifungal Activity

The controlled poisoning technique was performed [53] using potato dextrose agar (PDA; Bioxon, Becton Dickinson and Company, Querétaro, Mexico) supplemented with EOs at concentrations of 500, 1000, and 2000 µL/L. Tween 20 (1%) was added as an emulsifier to improve dispersion (Table 2). Mancozeb was included as a positive chemical control, allowing a direct comparison of the antifungal activity of the EOs (*C. zeylanicum*, *O. vulgare*, *S. rosmarinus*, and *P. nigrum*) with a conventional fungicide.

To determine the percentage of mycelial growth inhibition (RGI), 5 mm diameter PDA discs containing 10-day-old *C. gloeosporioides* (MA-L4) mycelium were placed at the center of each plate. EOs were applied at concentrations of 500, 1000, and 2000 µL/L, while distilled water at 500 µL/L was used as a negative control. All plates were sealed with Parafilm^®^ and incubated in darkness at 28 ± 2 °C for 15 days. Each treatment had four repetitions and an absolute witness without EOs, in duplicate. Finally, the RGI was calculated using the formula:

RGI (%) = [(DC − DT))/(DC − 5)] × 100 (1)

DC = Pathogen mycelial growth diameter in the control box.

DT = Pathogenic mycelial growth diameter in the presence of EOs treatment [54].

At the end of the incubation days, the sensitivity of the isolated was determined by means of a scale applied to plant extracts [50], modified to EOs (Table 3).

### 3.4. Fresh Fruit Severity Tests

To prepare the conidial suspension, 15-day-old *C. gloeosporioides* MA-L4 colonies grown on Petri dishes were scraped with a spatula in the presence of 5 mL of sterile distilled water containing Tween 20 (1 mL/L) to ensure adequate sporulation. From this mixture, 1 mL was taken to perform serial dilutions for accurate conidia counting using a Neubauer chamber. The suspension was stirred for 5 min to homogenize the conidia, and the final concentration was adjusted to 1 × 10^8^ propagules/mL. The prepared suspension is stored in refrigeration until its use [55].

Ripe fruits of Manila Mango variety (*Mangifera indica* L.) without visible symptoms of anthracnose were used, washed and the surface was disinfected by immersion in a 1% sodium hypochlorite solution for 3 min and then rinsed twice with sterile distilled water. They were then dried under a laminar flow hood. An emulsion was used as a coating and dispersion medium for essential oils (EOs). The emulsion was prepared with sterile distilled water (20 mL), Castalia Bio^®^ potassium soap (2.5 mL/L), and EOs at concentrations of 500, 1000, and 2000 µL/L, with the addition of Tween 20 (1 mL/L) [55]. Each fruit was immersed in the respective EO emulsion for 5 min and then dried at room temperature for 1 h. Fruits immersed only in sterile distilled water were used as a control treatment.

Subsequently, 2 mm deep wounds were performed in the middle part of the fruit with sterile needle and were inoculated with 20 µL of the conidial suspension (1 × 10^8^ propagules/mL). The fruits were incubated in wet chambers at 25 °C ± 2 for 15 days, observing the appearance and severity of injuries. Disease severity was analyzed using the scale reported by Corkidi et al. [56], with the IMAGEJ V.1.45 software (Table 4).

### 3.5. Statistical Analysis

The chemical composition of the EOs of *C. zeylanicum*, *O. vulgare*, *P*. *nigrum*, and *S. rosmarinus* was analyzed by an integral approach that combined statistical tools and visualization of data in R Studio-2022.12.0+353. It worked with a relative abundance matrix (%) of compounds identified by GC-MS, structured with compounds in rows and plant species in the columns. Subsequently, the compounds were classified into functional groups (monoterpenes, phenols, sesquiterpenes, among others) by inclusion of an additional categorical variable, which allowed to facilitate comparative analysis. To evaluate the chemical similarities between species, a hierarchical grouping analysis was applied using the HClust function, generating a dendrogram that represented relationships in terms of volatile composition. The visualization was carried out with the GGPLOT2 and Pheatmap packages, through which a colored matrix was coded to represent the relative abundance of the compounds and a graphic of stacked bar that showed the proportion of each functional group by species was generated. A color legend was integrated to facilitate the interpretation of predominant chemical groups. In addition, an additional bar chart was drawn that compared the number of compounds by functional class between species. To deepen the analysis of chemical diversity and overlap, a Venn diagram was built, identifying exclusive and shared compounds between species, in parallel, the UPSETT package was used to build an intersection chart (Upset Plot), from a binary arrangement of presence/absence, which represented in the X axis the combinations of species and in the axis and the number of compounds at each intersection. This tool complemented the Venn diagram, offering a more precise representation of chemical interrelations.

The results obtained in the pathogenicity and severity tests were analyzed using R Studio as a statistical platform. First, the normality of the data was evaluated by the Shapiro–Wilk test to ensure that they complied with the necessary assumptions for parametric analysis. Subsequently, the data that showed normal distribution was subjected to a two-way variance analysis (ANOVA) to determine significant differences between the treatments. Finally, the Tukey multiple comparisons test with a level of significance of *p* < 0.05 was applied to identify specific differences between means. Finally, a heat map associated with the post-harvest deterioration of mango fruits was used for the inhibition percentage by means of a red scale (0 to 100%), while the severity was represented by the size of the black bubbles superimposed on each cell. This approach allowed us to simultaneously interpret the antifungal efficacy and the severity of the damage under different concentrations and species of EOs.

## 4. Conclusions

The analysis of EOs of *C. zeylanicum*, *O. vulgare*, *P*. *nigrum*, and *S. rosmarinus* by GC-MS evidenced differentiated and highly representative chemical profiles, with retention times between 4.6 and 31.2 min. The identification of compounds covered more than 98% of the total content in *S. rosmarinus* and *P. nigrum*, 92% in *C. zeylanicum*, and approximately 89% in *O. vulgare,* indicating high efficiency in chemical characterization. In *C. zeylanicum*, the aromatic monoterpenoid eugenol (61.88%) as well as benzyl alcohol (14.44%) dominated the composition, while *S. rosmarinus* presented a profile based on oxygenated monoterpenes, highlighting eucalyptol (32.42%). *O. vulgare* showed a notable diversity with high levels of sabinene hydrate (21.68%) and carvacrol (6.75%), predominantly monoterpenes and oxygenated monoterpenes. For its part, *P. nigrum* exhibited a high content of methyl eugenol (46.28%) and eugenol (32.93%), both belonging to the group of aromatic monoterpenoids. The analysis also revealed shared compounds between species, such as eugenol, present both in *C. zeylanicum* and *P. nigrum*, and the 4-terpineol in *O. vulgare*, which suggests possible functional similarities and synergies in its biological application. These results reinforce the potential of EOs as versatile tools for pathogenic control, given their chemical wealth and functional diversity.

EOs have a significant inhibitory effect against the *C. gloeosporioides* fungus, causing anthracnosis in mango fruits. In vitro tests, the EOs of *C. zeylanicum* (Cinnamon) and *P. nigrum* (Black pepper) reached a 100% inhibition of the growth of the pathogen in their highest concentrations, evidencing their high sensitivity to these compounds. In addition, the results in vivo showed that all the EOs evaluated managed to reduce the growth of the pathogen by more than 90% in real conditions, even in those that showed less in vitro activity, such as *S. rosmarinus* (Rosemary).

These findings suggest that EOs constitute a natural and effective alternative for the control of anthracnosis post-harvest, reducing dependence on chemical fungicides and promoting more sustainable agricultural practices that contribute to improving the quality and durability of mango fruit.

## Figures and Tables

**Table 1:** Chemical composition of *P. nigrum*, *S. rosmarinus*, *C. zeylanicumm*, and *O. vulgare* essential oils.

KI^C^	RI^L^	Component	Class	Content of the Components (%)			
				*P. nigrum*	*S. rosmarinus*	*C. zeylanicumm*	*O. vulgare*
918	911	Thujene	BM	-	-	-	3.35
923	921	α-Pinene	BM	0.23	30.78	0.44	3.55
936	937	Camphene	BM	-	2.19	-	-
942	946	2,4-Thujadiene	BM	-	1.12	-	-
965	958	Benzaldehyde	ArM	-	-	0.81	-
965	964	β-Pinene	BM	-	-	-	4.57
983	979	1-Octen-3-ol	AH	-	-	-	3.77
992	992	β-Myrcene	AM	9.54	4.58	-	3.97
1011	1010	3-Carene	BM	-	-	-	1.70
1021	1021	*p*-Cymene	ArM	-	-	0.49	3.72
1024	1028	Limonene	MM	0.57	-	0.72	1.24
1027	1029	Eucalyptol	BM	4.33	32.42	1.39	---
1037	1037	β-ocimene	AM	0.75	-	-	1.89
1043	1041	Benzyl alcohol	ArM	-	-	14.44	-
1065	1064	*γ*-Terpinene	MM	-	1.93	-	4.61
1084	1088	α-Terpinolene	MM	-	1.35	-	2.77
1097	1096	Sabinene hydrate	BM	-	-	-	21.68
1101	1098	Linalool	AM	0.51	2.91	3.49	-
1137	1138	Camphor	BM	-	3.07	0.45	-
1164	1165	Borneol	BM	-	3.99	-	-
1162	1165	Benzyl acetate	ArM	-	-	1.64	-
1174	1177	4-Terpineol	MM	0.48	-	0.53	3.44
1188	1190	α-Terpineol	MM	-	-	-	1.59
1201	1200	Isocarveol	MM	1.91	-	-	-
1202	1204	Verbenone	BM	-	7.04	1.55	-
1222	1228	Thymol methyl ether	ArM	-	-	-	4.62
1228	1232	cis-Geraniol	AM	-	-	0.41	-
1292	1291	Terpinen-4-yl acetate	MM	-	-	-	3.55
1235	1259	*E*-Cinnamaldehyde	ArM	0.34	-	2.97	-
1244	1262	Isobornyl acetate	BM	-	1.75	-	-
1298	1292	Thymol	ArM	-	-	-	1.89
1304	1302	Carvacrol	ArM	-	-	-	6.75
1360	1359	Eugenol	ArM	32.93	-	61.88	-
1405	1410	Methyleugenol	ArM	46.28	-	-	-
1427	1417	β-Caryophyllene	BS	0.82	2.05	0.68	2.46
1441	1444	β-Cubebene	BS	-	-	-	1.68
1449	1445	Elemene	MS	-	-	-	2.23
1784	1762	Benzyl benzoate	ArM	-	-	1.92	--
Total Aliphatic hydrocarbons (AH)							3.77
Total Aliphatic monoterpenes (AM)				10.8	7.49	3.9	5.86
Total Monocyclic monoterpenes (MM)				2.96	3.28	1.25	17.2
Total Bicyclic monoterpenes (BM)				4.56	82.36	3.83	34.85
Total Aromatic monoterpenes (ArM)				79.55		84.15	16.98
Total Monocyclic sesquiterpenes (MS)							2.23
Total Bicyclic sesquiterpenes (BS)				0.82	2.05	0.68	4.14
Total compounds				98.69	95.18	93.81	85.03

RI^C^ Retention index calculated relative to a series of n-alkanes (C8–C20) on Elite 5-MS capillary columns. RI^L^ Retention Index of the literature. Identification of the components: Comparison of RI with published data and comparison of mass spectra with those listed in the NIST 02 and with published data. (-) Not determined.

**Table 2:** EOs evaluated in different concentrations alongside control and Mancozeb Treatments.

Eos	Concentration (µL/L)		
*Cinnamomum zeylanicum*	500	1000	2000
*Origanum vulgare*			
*Salvia rosmarinus*			
*Piper nigrum*			
Control (Distilled water)	500 µL/L		
Mancozeb (C_4_H_6_MnN_2_S_4_)	1000 µL/L		

**Table 3:** Scale for the sensitivity evaluation of *C. gloeosporioides* to the application of EOs.

Classification	Sensitivity Level
Low (L)	<40%
Intermediate (I)	40–75%
Sensitive (S)	>75%

**Table 4:** Scale for severity evaluation in mango fruits [56].

Severity Index	Present in Fruit (%)	Description
0	0	Healthy
1	≤25%	Traces (Chlorotic spots)
2	26–50%	Light (Dark injuries 1 to 5 mm in diameter)
3	51–75%	Moderate (Dark lesions greater than 6 mm in diameter)
4	>75%	Severe damage (Sunk dark injuries)

## Data Availability

Informed consent was obtained from all subjects involved in this study. Further inquiries can be directed to the corresponding author.

## Supplementary Materials

The following supporting information can be downloaded at: https://www.mdpi.com/article/10.3390/plants14213249/s1, Figure S1: Chromatograms obtained by GC-MS of essential oils extracted by steam drag from dry plant material: a) *C*. *zeylanicum* (leaves), b) *S. rosmarinus* (leaves), c) *O. vulgare* (leaves), and d) *P. nigrum* (fruit); Figure S2: EOs extracted by steam drag from dry plant material: a) *C*. *zeylanicum* (leaves), b) *S. rosmarinus* (leaves), c) *O. vulgare* (leaves), and d) *P. nigrum* (fruit); Figure S3: Proportions of functional groups identified in volatile compounds of different species studied.
