# Supplementary material for: Effect of Essential Oils on Postharvest Management of Anthracnose Associated with Colletotrichum gloeosporioides (Penz.) Penz & Sacc., in Mango
Source: Plants (Basel). 2025 Oct 23;14(21):3249. doi: 10.3390/plants14213249 (PMC12608393; doi:10.3390/plants14213249)
Supplement: Supplementary file 1 [file plants-14-03249-s001.zip › plants-3818386-supplementary.pdf]

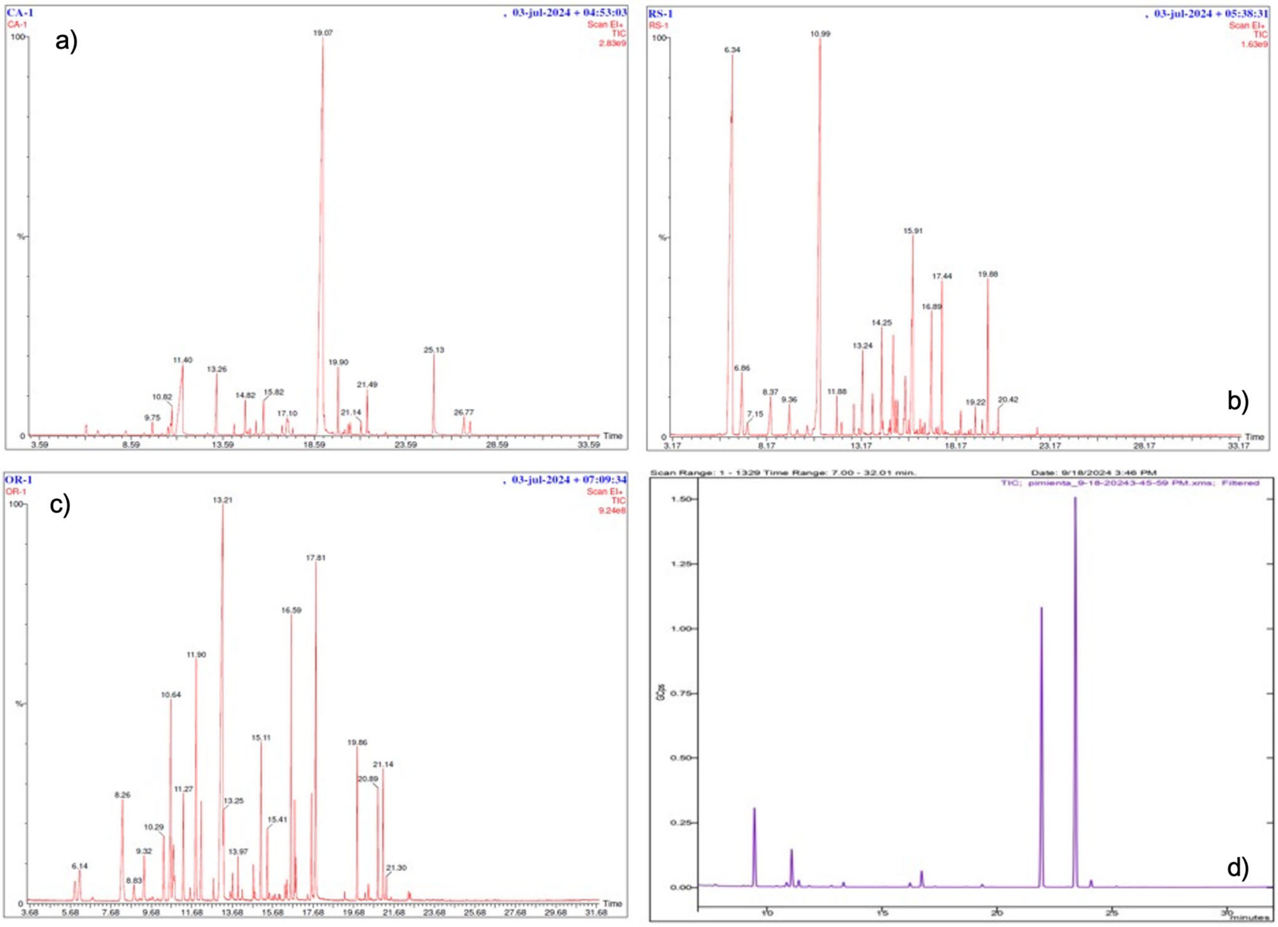

Figure S1. Chromatograms obtained by GC-MS of essential oils extracted by steam drag from dry plant material: a) *C. zeylanicum* (leaves), b) *S. rosmarinus* (leaves), c) *O. vulgare* (leaves), and d) *P. nigrum* (fruit).

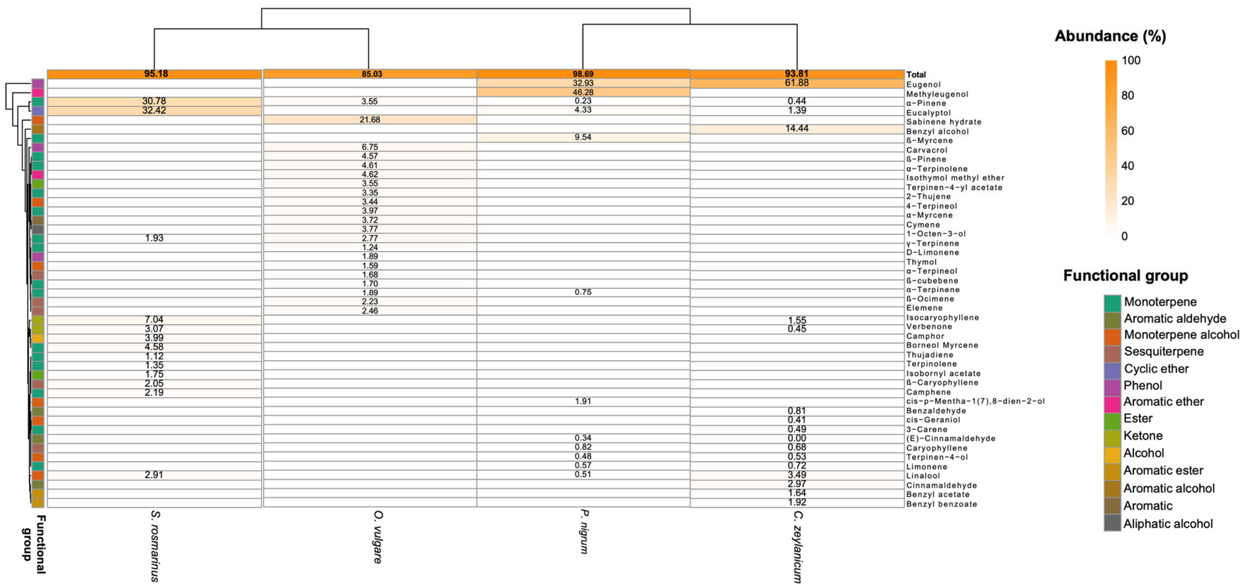

Figure S2. EOs extracted by steam drag from dry plant material: a) *C. zeylanicum* (leaves), b) *S. rosmarinus* (leaves), c) *O. vulgare* (leaves), and d) *P. nigrum* (fruit).

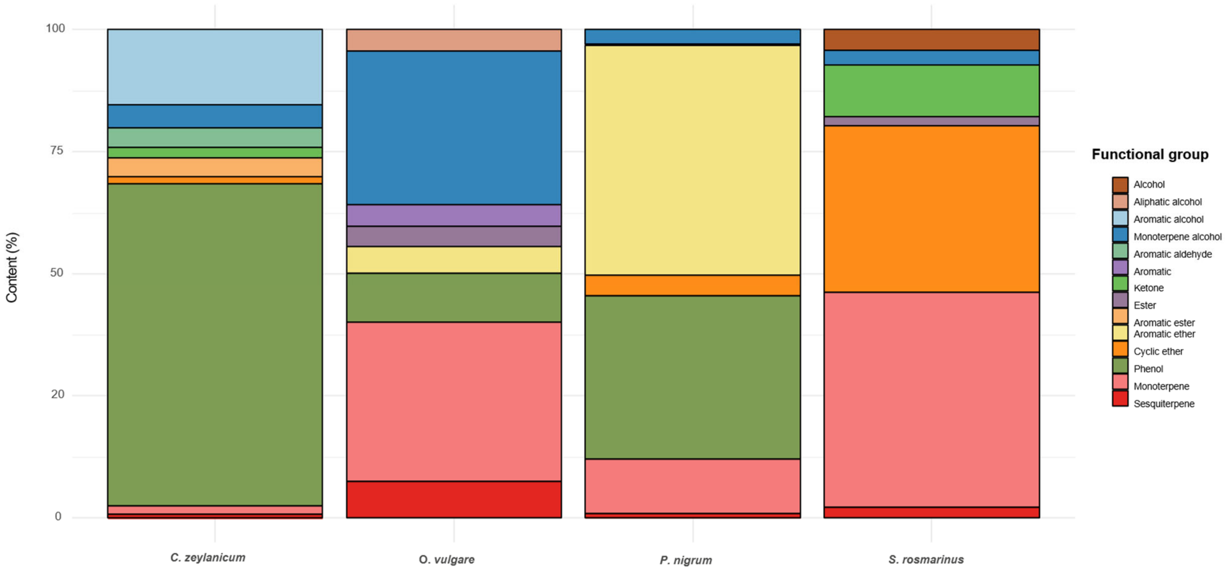

Figure S3. Proportions of functional groups identified in volatile compounds of different species studied.
